# Post-traumatic osteoarthritis development is not modified by postnatal chondrocyte deletion of *Ccn2*

**DOI:** 10.1242/dmm.044719

**Published:** 2020-07-14

**Authors:** Craig M. Keenan, Lorenzo Ramos-Mucci, Ioannis Kanakis, Peter I. Milner, Andrew Leask, David Abraham, George Bou-Gharios, Blandine Poulet

**Affiliations:** 1 Department of Musculoskeletal and Ageing Science, Institute of Life Course and Medical Sciences, University of Liverpool, William Henry Duncan Building, West Derby Street, Liverpool L7 8TX, UK; 2 College of Dentistry, University of Saskatchewan, Saskatoon, SK S7N 5E4, Canada; 3 Centre for Rheumatology and Connective Tissue Diseases, University College London, London NW3 2PF, UK

**Keywords:** Cartilage, CCN2, Osteoarthritis, Post-traumatic, Transgenic mouse, Trauma-induced

## Abstract

CCN2 is a matricellular protein involved in several crucial biological processes. In particular, CCN2 is involved in cartilage development and in osteoarthritis. *Ccn2* null mice exhibit a range of skeletal dysmorphisms, highlighting its importance in regulating matrix formation during development; however, its role in adult cartilage remains unclear. The aim of this study was to determine the role of CCN2 in postnatal chondrocytes in models of post-traumatic osteoarthritis (PTOA). *Ccn2* deletion was induced in articular chondrocytes of male transgenic mice at 8 weeks of age. PTOA was induced in knees either surgically or non-invasively by repetitive mechanical loading at 10 weeks of age. Knee joints were harvested, scanned with micro-computed tomography and processed for histology. Sections were stained with Toluidine Blue and scored using the Osteoarthritis Research Society International (OARSI) grading system. In the non-invasive model, cartilage lesions were present in the lateral femur, but no significant differences were observed between wild-type (WT) and *Ccn2* knockout (KO) mice 6 weeks post-loading. In the surgical model, severe cartilage degeneration was observed in the medial compartments, but no significant differences were observed between WT and *Ccn2* KO mice at 2, 4 and 8 weeks post-surgery. We conclude that *Ccn2* deletion in chondrocytes does not modify the development of PTOA in mice, suggesting that chondrocyte expression of CCN2 in adults is not a crucial factor in protecting cartilage from the degeneration associated with PTOA.

This article has an associated First Person interview with the first author of the paper.

## Abstract (precis)

**Summary:** Postnatal deletion of *Ccn2* in chondrocytes does not affect the development of post-traumatic osteoarthritis in mice.

## INTRODUCTION

Osteoarthritis (OA) is a major chronic degenerative disease of the joint, with limited therapies available to inhibit or slow disease progression. Mechanical trauma represents a major risk factor for OA, with mouse models of post-traumatic OA (PTOA) being widely used to study OA development. These include surgical models for development of different degrees of OA severity (Kamekura et al., 2005) and non-invasive repetitive joint trauma models, including the one established by Poulet et al. (2011). Both surgical and non-invasive models show similar hallmarks to those seen in human OA, including progressive articular cartilage (AC) degradation, subchondral bone sclerosis, osteophyte formation and synovial fibrosis and activation. In this study, we used both a surgical model and a non-invasive model to determine the role of the matricellular protein CCN2 in severe and moderate PTOA.

CCN2, formerly known as connective tissue growth factor (CTGF) (Perbal, 2018b), is a matricellular protein involved in key cellular functions including proliferation, adhesion and differentiation (Kubota and Takigawa, 2015), along with several complex biological processes including chondrogenesis (Perbal, 2004). Deletion of *Ccn2* during development demonstrated its essential role as a regulator of skeletal development by promoting endochondral ossification through the proliferation and differentiation of growth plate chondrocytes (Ivkovic et al., 2003), consistent with the ability of CCN2 to promote differentiation and proliferation of cultured chondrocytes.

CCN2 is expressed in adult AC, albeit at low levels (Tang et al., 2018; Nishida et al., 2004), and its expression increases significantly in OA chondrocytes (Omoto et al., 2004; Shimo et al., 2000; Takigawa et al., 2003). In addition, CCN2 promotes the expression of matrix genes and has been suggested as a potential cartilage repair factor (Nishida et al., 2004). Likewise, continuous cartilage-specific overexpression of CCN2 protects joints from age-related development of OA, highlighting a chondroprotective role of CCN2 (Itoh et al., 2013). Conversely, CCN2 overexpression in the synovial lining of mouse knee joints causes transient fibrosis and cartilage damage (Blaney Davidson et al., 2006). Moreover, mice in which CCN2 was globally deleted postnatally were protected from surgically induced OA; this effect was linked to the ability of CCN2 to activate latent TGFβ (also known as TGFB1-3) (Tang et al., 2018). These divergent results are consistent with the fact that CCN2, as a matricellular protein, has cell type- and context-dependent effects (Perbal, 2018a); it can promote the expression of extracellular matrix genes in chondrocytes or promote fibrosis/OA depending on the context. The elucidation of the precise role of CCN2 in joint health and OA, chondroprotective versus profibrotic/pro-osteoarthritic, needs to be explored further genetically. Therefore, to gain a better understanding of the role of CCN2 in adult cartilage, we exposed chondrocyte-specific *Ccn2* knockout mice to two distinct models of PTOA.

## RESULTS

### *Ccn2* was successfully deleted in chondrocytes in adult mice

**Fig. 1.**
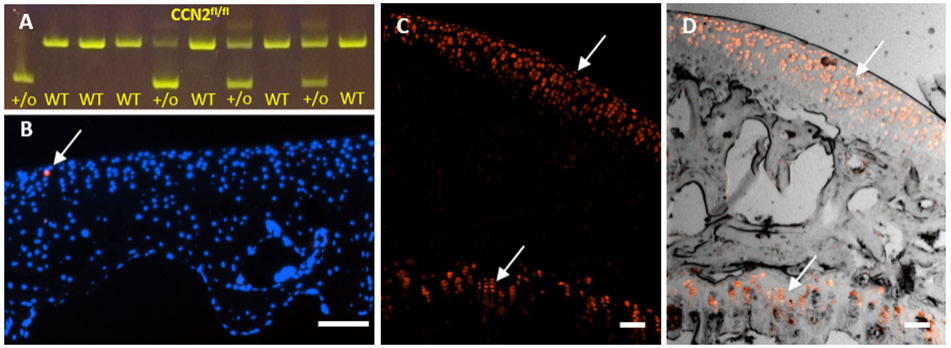
**Confirmation of Cre recombination and *Ccn2^fl/fl^* transgene deletion in chondrocytes in −30 kb *Acan* CreER^T2^ adult mice.** (A) Genotyping from tail tips, the genomic DNA from WT and *Ccn2* Cre^+/o^ mice showed the presence of the CCN2 flox product in all mice (first band) and the additional lower band in Cre^+/o^ mice only, (+/o) ∼500 bp in size, confirming Cre recombination and deletion of the CCN2 floxed product. (B-D) Histological images of the tibial epiphysis in *Ccn2* Cre^+/o^ crossed with the tdTomato reporter mouse 4 weeks after the last corn oil control injection (B) or tamoxifen injection (C,D). Corn oil treatment resulted in no recombination (except one single chondrocyte stained red, arrow in B; blue staining of nuclei with Hoechst stain). (C,D) Tamoxifen treatment resulted in expression of tdTomato fluorescence (red) in chondrocytes throughout the AC and growth plate (top and bottom arrows, respectively). Scale bars: 100 µm.

To verify that deletion of *Ccn2* in chondrocytes had occurred in response to tamoxifen injection, a tail tip containing ACAN-expressing cells from the intervertebral disc and endplates was taken post-cull from each individual mouse, and PCR was performed on extracted genomic DNA for genetic recombination and exon deletion. All mice contained a 1000 bp band corresponding to the floxed *Ccn2* allele, and Cre^+/o^ mice contained an additional band at ∼500 bp corresponding to the allele generated by Cre recombination resulting in the Cre-mediated deletion of *Ccn2* (Fig. 1). Cre^WT^ mice showed no recombination. To validate the efficiency of the CreER^T2^ system and the subsequent deletion of the transgene in these new *Acan* −30 kb CreER^T2^ mice, *Ccn2^fl/fl^* with Cre^+/o^ (*Ccn2* KO) mice were crossed with a tdTomato reporter mouse, which enabled detection of recombined CreER^T2^ via fluorescence, after administration of tamoxifen to the mice. In control corn oil-treated mice, no tdTomato was seen in the whole joint cells (only a chondrocyte was positive; Fig. 1), demonstrating that no recombination of CreER^T2^ occurred. After tamoxifen treatment, there was clear recombination of CreER^T2^ as evident by the intense expression of tdTomato protein in all chondrocytes located in both the AC and the growth plate (Fig. 1).

### Conditional *Ccn2* deletion in chondrocytes does not prevent the development of OA in a non-invasive loading model of trauma-induced OA

**Fig. 2.**
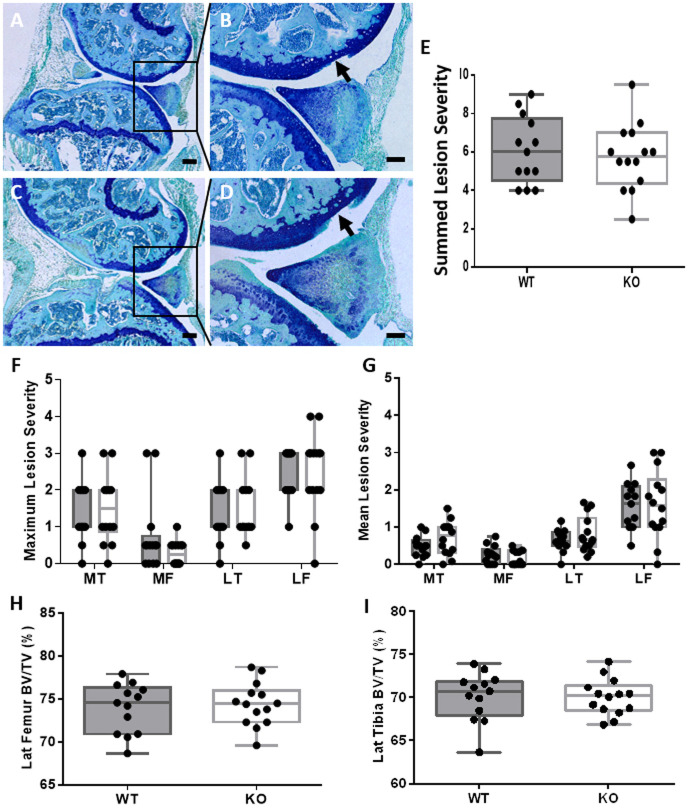
**Deletion of *Ccn2* in chondrocytes does not prevent OA development in a non-invasive loading model of PTOA.** (A-D) Toluidine Blue-stained sections of the tibiofemoral joint of WT (A,B) and *Ccn2^fl/fl^* KO (C,D) mice showing development of AC lesions localized to the lateral femur in loaded knee joints (arrows) 6 weeks after the last loading episode. (E-G) AC lesion severity scores across the whole knee joint showing no differences between WT and *Ccn2^fl/fl^* KO mice in summed maximum scores (E) or in maximum (F) and mean (G) lesion severity scores for each joint compartment. (H,I) BV/TV of the epiphyseal bone in the lateral (Lat) femur (H) and lateral tibia (I) showed no significant difference in bone structure between WT and *Ccn2^fl/fl^* KO. Whiskers represent minimum and maximum values, with box for interquartile range with median, and showing individual animals. LF, lateral femur; LT, lateral tibia; MF, medial femur; MT, medial tibia. Scale bars: 200 µm in A,C; 100 µm in B,D. Grey boxes, WT; white boxes, *Ccn2^fl/fl^* KO. Number of animals: *n*=13 WT and *n*=14 *Ccn2* KO. Statistical test performed: Mann–Whitney *U*-test and Wilcoxon signed-rank tests for lesion severity, and unpaired Student's *t*-test for bone measurements.

Mice [*Ccn2* KO and wild type (WT)] were treated with tamoxifen then mechanically loaded to induce PTOA. Histological examination of the loaded limb in both WT (*n*=13) and *Ccn2* KO (*n*=14) mice showed the development of OA lesions, with loss of hyaline AC and exposure of the articular calcified cartilage primarily in the lateral femur (Fig. 2). Assessment of the severity of the lesions in each compartment throughout the entire tibiofemoral joint (medial and lateral, tibia and femur) showed no significant differences in the AC lesion mean, maximum and summed severity scores between WT and *Ccn2* KO mice. Micro-computed tomography (micro-CT) analysis of the epiphyseal bone of the lateral femur and tibia showed no differences in bone volume/tissue volume (BV/TV) between WT and KO (Fig. 2). Together, these data indicate that deletion of *Ccn2* postnatally from ACAN-expressing chondrocytes had no effect on the development of OA in a non-invasive model of moderate PTOA.

### *Ccn2* deletion specifically in chondrocytes does not prevent the development of OA in a surgical model of severe PTOA

**Fig. 3.**
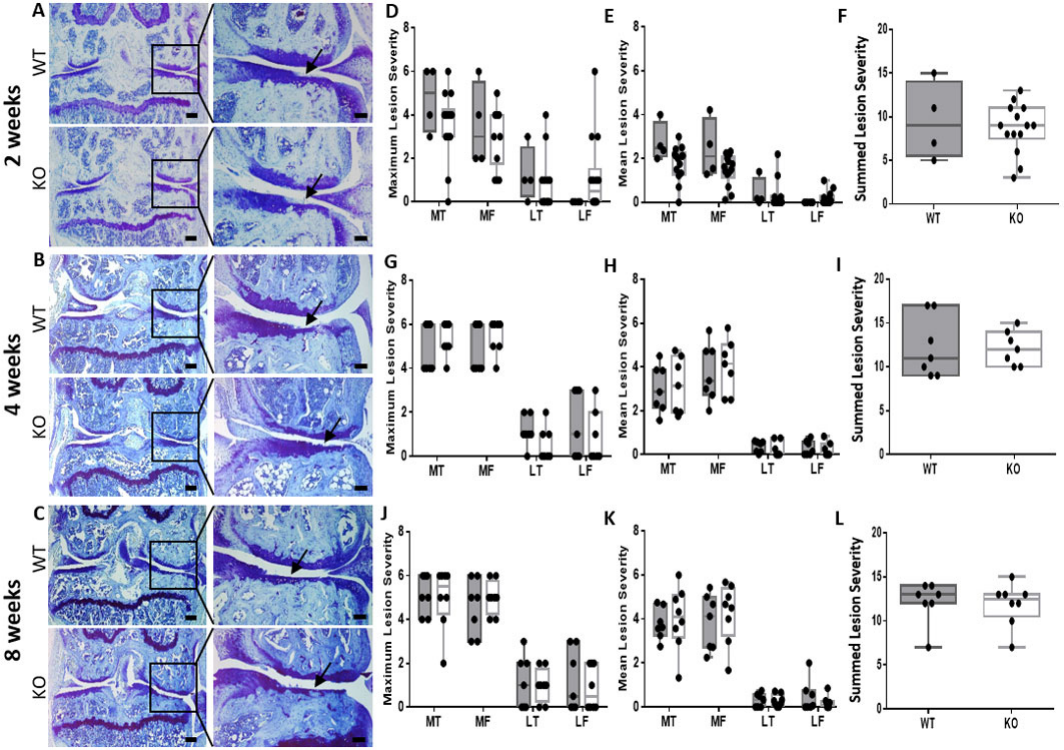
**Deletion of *Ccn2* in chondrocytes does not mitigate AC lesion severity in a surgical model of severe PTOA.** (A-C) Toluidine Blue-stained sections from WT and *Ccn2^fl/fl^* KO mice 2, 4 and 8 weeks post-surgery showing development of OA on the medial tibia (arrows) in both WT (top) and *Ccn2^fl/fl^* KO (bottom). (D-L) Maximum and mean lesion severity in each individual joint compartment and summed maximum scores showing no significant difference between OA severity in WT and *Ccn2^fl/fl^* KO mice at 2 weeks (D-F), 4 weeks (G-I) and 8 weeks (J-L) post-surgery. Whiskers represent minimum and maximum values, with box for interquartile range with median, and showing individual animals. LF, lateral femur; LT, lateral tibia; MF, medial femur; MT, medial tibia. Scale bars: 200 µm in left column (A-C); 100 µm in right column (A-C). Grey boxes, WT; white boxes, *Ccn2^fl/fl^* KO. Number of animals: 2 weeks, *n*=4 WT and *n*=14 KO; 4 weeks, *n*=7 WT and *n*=7 KO; 8 weeks, *n*=7 WT and *n*=8 KO. Statistical test: Mann–Whitney *U*-test test and Wilcoxon signed-rank test.

To confirm that deletion of *Ccn2* in chondrocytes of adult mice had no effect on OA, a second model of PTOA was used. This model incorporated a different Cre (*Acan* −10 kb CreER^T2^), which had been shown previously to be expressed in articular chondrocytes (Han and Lefebvre, 2008). At 2 weeks post-surgery, moderate AC lesions were already visible in the medial compartment of both WT and *Ccn2* KO mice, with exposure of the underlying calcified cartilage (Fig. 3). At 4 weeks post-surgery, the damage was extensive, with both WT and *Ccn2* KO mice suffering substantial loss of AC in the medial compartment of the tibiofemoral joint. By 8 weeks post-surgery there was widespread, significant damage across the entire medial side of the tibiofemoral joint in both WT and *Ccn2* KO mice, including near-complete loss of AC. Cartilage lesion scoring showed no significant differences between WT and *Ccn2* KO at all time points.

## DISCUSSION

The aim of this study was to determine the postnatal role of CCN2 in AC during PTOA in two different models. We have found that despite its importance during skeletal development and its increased expression by OA chondrocytes, CCN2 in AC chondrocytes in adult joints did not play a significant role in preventing PTOA development.

A number of studies have focused on the regenerative ability of CCN2 owing to its role in cellular proliferation and differentiation during chondrogenesis (Takigawa et al., 2003; Shimo et al., 2000). Indeed, it has been shown *in vitro* that CCN2 induces chondrocyte proliferation and differentiation, with increased synthesis of cartilage matrix proteins, including proteoglycans, ACAN and collagen types II and X (Shimo et al., 2000; Fujisawa et al., 2008). Subsequently, it was found that CCN2 plays a role in chondrogenesis during fractured bone healing; CCN2 expression in this process responds to MAPK and retinoid receptor gamma signalling pathways (Shimo et al., 2019, 2005, 2020; Nakata et al., 2002; Yosimichi et al., 2006). CCN2 expression is highly regulated by TGFβ (Song et al., 2007), with its promoter transcriptional activity maintained by a TGFβ response element (Eguchi et al., 2001). The importance of CCN2 in chondrogenesis can be seen in the severe developmental phenotype in mice lacking CCN2; indeed, Ivkovic et al. (2003) showed impaired chondrocyte proliferation and matrix deposition in the hypertrophic zones of growth plate cartilage linked to defective matrix remodelling and growth plate angiogenesis, concurrent with decreased vascular endothelial growth factor (VEGF). In parallel, overexpression of CCN2 in chondrocytes led to enhanced endochondral ossification, with elongated bones, increased proteoglycans and collagen II content in newborn transgenic mice, mediated in part by enhanced accumulation of insulin-like growth factors (IGFs) (Tomita et al., 2013). In adults, CCN2 overexpression was shown to be chondroprotective, by inhibiting ageing-induced development of OA, with increased adult AC matrix synthesis and decreased matrix degrading enzymes (Itoh et al., 2013). Its role in chondrogenesis highlighted its potential as a possible regenerative therapy for the treatment of OA, particularly given that exogenously added recombinant CCN2 did not stimulate hypertrophy of chondrocytes *in vitro* (Nishida et al., 2002). Moreover, treatment of AC defects with recombinant CCN2 showed repaired cartilage to be structurally similar to healthy AC (Nishida et al., 2004).

A study by Tang et al. (2018) showed that a postnatally induced global deletion of *Ccn2* caused a thickening of cartilage that resulted in protection from ageing-induced OA. Deletion of *Ccn2* from all cells was induced in adult mice, and post-traumatic surgical OA induction resulted a decrease in AC degradation, increased cartilage thickness and activation of canonical TGFβ signalling SMAD2 phosphorylation. Their study also showed that CCN2 was involved in the regulation of TGFβ activation by sequestration of its latent form in the matrix and activation of the cell surface receptor TGFβR3. The authors suggested that in global *Ccn2* deletion, chondrocytes might be responding to increased soluble latent TGFβ from lack of sequestration in the cartilage matrix or produced by other non-AC tissues. To test whether this phenotype is induced primarily by cartilage or by other joint tissues, we selectively deleted *Ccn2* in chondrocytes in adult mice and assessed post-traumatic OA development. The lack of effect of chondrocyte-specific compared with global deletion of *Ccn2* in mice (which protected from development of OA) suggests that CCN2 expression in other tissues, including those surrounding the tibiofemoral joint, such as synovium and epiphyseal bone, might be required for protection from OA. In addition, it has been shown that CCN2 can be found in serum and synovial fluid (Honsawek et al., 2012; Yang et al., 2017), which might compensate the absence of CCN2 in chondrocytes in the present study.

CCN2 is expressed by a number of other cells/tissues in the joint, such as synovial fibroblasts (Blaney Davidson et al., 2006), and has been shown to induce hallmarks of OA such as synovial fibrosis, cartilage degeneration and osteophyte formation. It is also entirely feasible that the lack of effect seen in chondrocyte-specific deletion in our studies is linked to secretion of CCN2 from these other joint tissues and its release into the joint space, thereby preventing any effects of chondrocyte-specific deletion on OA development from being observed. It has been shown that fibrosis is highly linked to TGFβ-induced CCN2 expression and that these tissues can also express high levels of matrix degrading enzymes (such as MMPs) (Blaney Davidson et al., 2006). This would therefore suggest that pathological non-AC joint tissues, such as synovial fibrosis, are controlled by CCN2 expression during post-traumatic OA development and contribute to AC degradation.

The effects of *Ccn2* deletion on chondrogenesis and OA development are similar to those of other CCN members. Indeed, deletion of *Ccn4*, also known as *Wisp1*, was shown to promote chondrogenesis and cartilage repair (Yoshioka et al., 2016), whereas it can also promote OA development (van den Bosch et al., 2017). The latter was linked to differential effects of *Ccn4* deletion on OA tissues, with synovial tissue being more responsive to *Ccn4* deletion than cartilage in OA joints. These data therefore suggest a protective effect of CCN4 in cartilage, but a pro-arthritogenic role via synovial fibrosis. Similar mechanisms could also be involved in *Ccn2* deletion, whereby its protective role focuses on chondrogenesis (during development and facture repair) and its pathological role targets mainly fibrotic events (synovial fibrosis), therefore explaining our lack of effects of *Ccn2* deletion in chondrocytes.

Other cells, including those derived from mesenchymal stem cells (MSCs) and MSC-like progenitor cells, might also have affected the levels of CCN2 in the joint. These cells are present in all joint tissues and can differentiate into several populations of cells, including those with chondrogenic potential (Barry and Murphy, 2013). These cells, which are not targeted for *Ccn2* deletion, might have been recruited in response to lesion formation, leading to increased expression of CCN2 in the joint and subsequent loss of the KO effect on OA development.

It should be noted that the absence of any observable responses in our studies is not a result of insufficient Cre recombinase activity and the subsequent deletion of *Ccn2* from chondrocytes. The efficiency of Cre recombinase activity for the *Acan* −10 kb enhancer had been tested previously and shown to be effective (Cascio et al., 2014), and the new −30 kb enhancer showed a proportionally greater number of chondrocytes expressing the transgene (Li et al., 2018). To confirm the efficiency of this new *Acan*-specific CreER^T2^ system, *Acan* −30 kb CreER^T2^ with *Ccn2^fl/fl^* mice were crossed with tdTomato reporter mice (Fig. 2), which allowed for detection of Cre recombinase activity following tamoxifen injection by visualisation of the tomato fluorescence compared with oil-injected control mice, and confirmed recombination, hence CCN2 deletion, from all AC chondrocytes. Furthermore, the use of two different *Acan* Cre systems to drive Cre recombinase expression in chondrocytes ensured that any responses were independent of Cre activity targeting and were most likely to be a direct result of the action of *Ccn2* deletion.

In the present study, we used two models of PTOA, with different severities of OA progression. The non-invasive loading of the knee has previously been shown to induce moderate OA lesions on the lateral femur (Poulet et al., 2011), whereas surgical intervention is known to lead to a higher degree of OA severity. The importance of using different models pertains to the fact that, although similar pathologies can be seen in both models, both can trigger different cellular responses linked to the severity of the disease. For example, the surgical model might trigger more severe inflammatory responses. In addition, the mechanical environment is severely affected in the surgical model throughout the whole study, whereas the traumatic loads are applied at specific and controlled times, with a maximum of six episodes of 7 min; the rest of the time, the mechanical environment is relatively normal compared with that engendered by surgical instability. In future studies, the lack of effects of chondrocyte-specific CCN2 expression in adults could be tested in other models of OA, including ageing- and obesity-induced OA.

In conclusion, this study showed, through the use of two models of PTOA, that CCN2 expression by chondrocytes is not required for maintenance of cartilage in adults. Further work will confirm whether CCN2 expression by other non-cartilage tissues within the joint might be involved in development of PTOA.

## MATERIALS AND METHODS

### Animals

All work was carried out in accordance with the UK Home Office guidelines and regulations under the Animals (Scientific Procedures) Act 1986. All mice (C57CBA background) were housed in the specific pathogen-free biological services unit at the University of Liverpool, UK and housed in cages of up to five mice, with 12 h-12 h light-dark cycle and *ad libitum* food and drink. Using chondrocyte-specific *Acan* enhancers, two conditional *Ccn2* KO mouse models were generated. The first contained an *Acan* −10 kb CreER^T2^ enhancer, as described by Han and Lefebvre (2008). This produced *Acan* −10 kb CreER^T2^×*Ccn2^fl/fl^* mice, in which *Ccn2* was deleted from articular chondrocytes. The second contained an *Acan* −30 kb CreER^T2^ enhancer described by Li et al. (2018). This produced *Acan* −30 kb CreER^T2^×*Ccn2^fl/fl^* mice, in which *Ccn2* was deleted from all chondrocytes. Deletion of *Ccn2* was regulated using a tamoxifen-inducible CreER^T2^ in adult mice, to avoid effects of *Ccn2* deletion during development and growth. For visualisation of the Cre recombinase efficiency in mouse knee joints, Rosa26-loxP-STOP-loxP-tdTomato (R26R-tdTomato; JAX7914) were mated with *Acan* −30 kb CreER^T2^ mice.

### Tamoxifen induction of *Acan* CreER^T2^

Before the start of all *in vivo* experiments, deletion of *Ccn2* or STOP in reporter tdTomato using *Acan* CreER^T2^ was induced at 8 weeks of age using tamoxifen (Sigma-Aldrich, UK). All mice, whether WT or *Ccn2^fl/fl^*, were administered tamoxifen intraperitoneally at a dose of 1 mg/10 g body weight on days 1, 3 and 5 and were weighed before injection on each day. After tamoxifen injections, mice were left for 1 week before any experimental work commenced. The tdTomato mice were sacrificed 4 weeks after last tamoxifen injection.

### Non-invasive mechanical loading model of PTOA

Right knees of 10-week-old male *Acan* −30 kb CreER^T2^×*Ccn2^fl/fl^* [*n*=13 (Cre^WT^), *n*=14 (Cre^+/o^)] mice were loaded non-invasively, using a model previously described (Poulet et al., 2011) to induce PTOA. Briefly, the mouse was anaesthetised, and the right hindlimb was placed in a custom-made cup, with the knee in flexion. A peak load of 9 N was applied for 0.05 s, with a rise and fall time of 0.025 s and a baseline hold time of 9.9 s for 40 cycles. A baseline load of 2 N was applied to keep the tibia in place during peak loading. All mice were subjected to this pattern three times per week for 2 weeks. Loading was performed using an ElectroForce 3100 (TA Instruments, USA). Mice were weighed after each loading episode, and on a weekly basis after completion of the loading regimen. All mice were sacrificed 6 weeks post-loading and samples prepared for micro-CT and histological analysis.

### Surgical model of PTOA

Left knee joints of 10-week-old *Acan* −10 kb CreER^T2^×*Ccn2^fl/fl^* mice underwent surgical transection of the medial meniscus (MM) and the medial meniscotibial ligament (MMTL). Mice were induced and maintained under a plane of general anaesthesia using isoflurane during the surgical procedure. A small incision was made over the medial aspect of the patella tendon and the joint capsule incised. Using blunt dissection, small amounts of fat were removed to permit visualisation of the MM and MMTL. Using a scalpel, the MM and MMTL were transected using an upwards motion from the cranial horn of the MM on the proximal tibial plateau. Once transected, the joint capsule and the skin were sutured. Mice were immediately transferred to a heated postoperative recovery room. They were monitored daily to ensure that they were in good health. Mice were sacrificed 2 weeks postoperatively [*n*=4 (Cre^WT^), *n*=14 (Cre^+/o^)], 4 weeks postoperatively [*n*=7 (Cre^WT^), *n*=7 (Cre^+/o^)] and 8 weeks postoperatively [*n*=7 (Cre^WT^), *n*=8 (Cre^+/o^)]. Samples were prepared for micro-CT and histological analysis.

### Specimen preparation

All animals were sacrificed by cervical dislocation. Experimental and contralateral joints were dissected, immediately fixed in 10% neutral buffered formalin for 24 h and transferred to 70% ethanol for storage.

### Micro-CT analysis

**Fig. 4.**
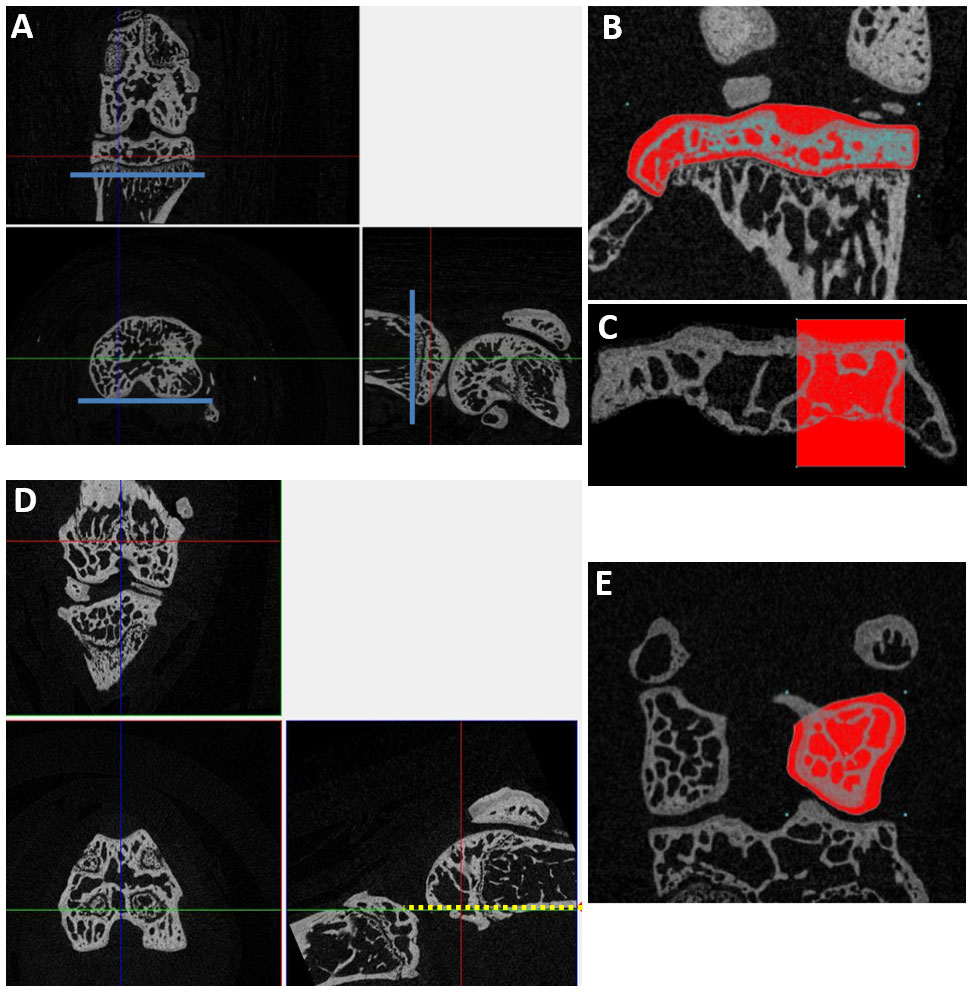
**Description of the method for the micro-CT analysis of the knee joint epiphyseal bone.** (A) Tibial reorientation in DataViewer (Bruker microCT; showing the three planes of view: top, coronal; bottom left, transaxial; bottom right, sagittal), with the growth plate and posterior edge of the tibia aligned in the different views along the blue line. (B,C) Manual drawing of the region of interest (red) in the tibial epiphysis (B) and selection of the lateral compartment (C) in CTAn (Bruker microCT). (D) Femur orientation in DataViewer, with the shaft of the femur aligned with the dotted yellow lines. The region of interest will then be confined to the posterior aspect of the joint (below the yellow dotted line). (E) Drawing of the region of interest in CTAn to include the whole lateral condyle of the femur.

Experimental and contralateral joints from the non-invasive mechanical loading model were scanned at a resolution of 4.5 µm using a 0.25 mm aluminium filter, with a rotation step of 0.6° (Skyscan 1272; Bruker microCT, Belgium). Image reconstruction was performed using NRecon software (Bruker microCT, Belgium), followed by manual selection of regions of interest for the tibial and femoral epiphyses. Datasets were first oriented in DataViewer (Bruker microCT) to ensure that all samples were analysed in the same orientation depending on the structure analysed (Fig. 4A,E). Newly orientated samples were opened in CTAn (Bruker microCT) and regions of interest drawn to include the lateral condyles; these included the epiphysis delimited by the joint space and the growth plate, and the edges of the articulating surface for each condyle. The BV/TV ratio was determined in CTAn. Data were tested for normality, and unpaired Student's *t*-test was used for statistical evaluation, with significance set at *P*<0.05, comparing osteoarthritic WT and *Ccn2^fl/fl^* KO joints.

### Histological analysis

Samples were decalcified in either 10% EDTA (Sigma-Aldrich, UK) for 2 weeks or 10% formic acid (Sigma-Aldrich, UK) for 1 week. Once decalcified, samples were given a processing number independent of their genotype, processed, paraffin embedded in either the coronal (surgical model) or sagittal plane (loading model), and 6-µm-thick sections were taken throughout the entire joint. Sections across the joint at 120 µm intervals were stained with Toluidine Blue/Fast Green (0.04% in 0.1 M sodium acetate buffer, pH 4.0), and cartilage lesion severity was graded using the Osteoarthritis Research Society International (OARSI) histopathology initiative scoring method (Glasson et al., 2010). Grading each of the four compartments of the tibiofemoral joint (lateral and medial tibia and femur) throughout the entire joint allowed for the determination of a maximum lesion grade (most severe lesion) for the whole joint and each individual compartment. The mean score, which involved determining the average grade across multiple slides, was calculated for each joint and each compartment (Poulet et al., 2011). The summed score was determined by adding together the maximum score of each compartment per joint. Osteophyte formation was graded histologically using the scale described by Kamekura et al. (2005). Statistical analysis was performed using the Mann–Whitney *U*-test and the Wilcoxon signed-rank test. Data are presented as box plots of interquartile range, median, minimum and maximum, showing all individuals.

### Cryosectioning

Samples from tdTomato were dissected, fixed in 4% paraformaldehyde at 4°C for 24 h, decalcified in 10% EDTA for 2 weeks, embedded using OCT embedding media (Tissue-Tek, Sakura Europe) in either the coronal (WT) or sagittal (Cre^+/o^) plane, and stored at −80°C until required. Sections were taken at 5 µm thickness until the middle of the joint was reached. A section showing the entire tibiofemoral joint was then collected and stained with Hoechst stain for 30 min. Images were obtained using a Zeiss Axio Observer apotome microscope (Zeiss, Germany).
